# Implementing an education program for nurse-midwives focused on early essential care for breast milk expression among mothers of preterm infants

**DOI:** 10.1186/s13006-021-00395-z

**Published:** 2021-06-26

**Authors:** Rie Tanaka, Shigeko Horiuchi

**Affiliations:** 1 Graduate Course of Midwifery, Teikyo University, 2-11-1 Kaga, Itabashi-ku, Tokyo, 173-8605 Japan; 2 St. Luke’s International University, 10-1 Akashi-cho, Chuo-ku, Tokyo, 104-0044 Japan

**Keywords:** Preterm infants, Mothers, Breast milk expression, Obstetric nursing, Education

## Abstract

**Background:**

Although breastfeeding guidelines for infants admitted to the neonatal intensive care unit (NICU) have been introduced in Japan, these guidelines have not been reflected in practice. To improve this situation, it is important for nurses and nurse-midwives to acquire knowledge of appropriate care. This study examined changes in nurse-midwives’ knowledge, attitude, and implementation of appropriate care after implementing an education program focused on early essential care for breast milk expression among mothers of preterm infants.

**Methods:**

This pre- and post-intervention study using a single group was conducted from June 2018 to May 2019 and enrolled 36 nurse-midwives in one perinatal medical center. The education program content included nurses’ roles in early essential care for milk expression and the necessary care to promoting breast milk production among mothers of preterm infants. The nurse-midwives’ knowledge and attitude on care were investigated 3 months before (pre-1), just before (pre-2), just after (post-1), and 3 months after (post-2) the program. The nurse-midwives’ care implementation was investigated at pre-1, pre-2, and post-2. During this time, 11 mothers (before: 7, after: 4) reported the status of milk expression for 10 days after birth.

**Results:**

The *mean knowledge scores* of the nurse-midwives at post-1 and post-2 were significantly higher than that at pre-2 (post-1: *p* <  0.001, post-2: *p* <  0.001). The *attitude on care scores* at post-1 and post-2 were significantly higher than that at pre-2 (post-1: *p* < 0.001, post-2: *p* = 0.010). The *care implementation score* at post-2 was significantly higher than that at pre-2 in eight items (e.g., Q7 Explain about the effect of initiating milk expression early and assist mothers in it). However, the education program did not cause any changes in the mothers’ *initiation and frequency of milk expression*, and *breast milk volume* after birth.

**Conclusions:**

The significant increases in the knowledge, attitude on care, and care implementation scores of the nurse-midwives suggest the beneficial effects of the education program. The small number of mothers in the survey on the practice of breast milk expression limited the full determination of the benefits of the education program for nurse-midwives.

**Supplementary Information:**

The online version contains supplementary material available at 10.1186/s13006-021-00395-z.

## Background

Preterm birth remains a major global problem with an estimated rate of 10.6% across the world in 2014 [1]. In Japan, the proportion of preterm birth has been increasing from 4.1% in 1980, 5.4% in 2000, to 5.7% in 2017 despite the decreasing number of births [2]. Children who do not receive breast milk have increased risk of infectious diseases, being overweight, having diabetes, and lower intelligence [3]. This indicates that preterm mothers should know how to initiate milk expression in the early postpartum period.

The international breastfeeding guidelines recommend supporting all mothers in initiating breastfeeding within 1 h after birth, and coaching mothers who are separated from their infants on how to express breast milk [4]. The Japanese guidelines recommend initiating milk expression within 6 h after birth [5], which is not completely the same as the international breastfeeding guidelines. Previous studies have reported on the appropriate timing of initiating milk expression (i.e., within 1 h after birth) [6, 7]. As for the Japanese guidelines, the timing for initiating milk expression is based on the evidence presented in the study by Furman et al. [8]. As the Japanese guidelines have never been reviewed since their publication in 2010, the guidelines should be updated in line with international standards.

Currently, some Japanese preterm mothers fail to follow the Japanese guidelines as they do not initiate milk expression at the appropriate timing [9, 10] and they do not express breast milk frequently [9–11]. As a result, their breast milk volume remains small [9, 10]. Some Japanese mothers who have given birth to a preterm infant have not been aware of their own ability to produce breast milk until receiving support from nurses and nurse-midwives, and they have struggled with continuing to produce milk [12].

The Japanese guidelines describe actions to be enforced in the NICU. Thus, these guidelines may not be fully disseminated to nurses and nurse-midwives in the maternity ward who play a very important role in initiating milk expression of preterm mothers. In fact, nurse-midwives in the maternity ward were previously found to have no clear standards on care for initiating and continuing milk expression, and they took care of mothers without fully understanding their milk expression situation [10]. When nurses and nurse-midwives in the maternity ward provide appropriate care to mothers of preterm infants in relation to milk expression, mothers are anticipated to initiate milk expression at the earliest time possible to promote breast milk production. Milk output in the first week after birth was found to be significantly predictive of the milk output in the sixth week [13]. Therefore, early initiation of milk expression helps maintain a sufficient breast milk volume until the infants are sufficiently strong to latch on and suckle the breast.

Continuing education on breastfeeding improves the knowledge and clinical practice of nurses and nurse-midwives [14]. Therefore, it is crucial for nurses and nurse-midwives to fully understand their own roles in breastfeeding support for mothers of preterm infants and to acquire comprehensive knowledge of appropriate care for milk expression to improve breastfeeding.

## Methods

### Purpose

The main aim of this study was to examine changes in the knowledge, attitude, and implementation of appropriate care of nurses and nurse-midwives after implementing an education program for nurses and nurse-midwives focused on early essential care for breast milk expression among mothers of preterm infants.

The primary outcome was knowledge of appropriate care related to breast milk expression of nurses and nurse-midwives. The secondary outcomes were attitude and care implementation of nurses and nurse-midwives. Evaluation of the education program was conducted by nurses and nurse-midwives. We also investigated if education for nurses and nurse-midwives would change the status of milk expression among mothers of preterm infants.

### Study design

The study used a pre- and post-intervention design in a single group of nurses and nurse-midwives. An observational study was also conducted to examine changes in the status of milk expression among mothers of preterm infants in the period of inquiry before and after the education program for nurses and nurse-midwives.

### Setting

The setting was a perinatal medical center which had 33 beds in the maternity ward, six beds in the NICU, and 10 beds in the growing care unit. The perinatal medical center was located in a large urban area. The center has more than 1000 births per year. There are about 50 nurses and nurse-midwives who work in the maternity ward, with most being nurse-midwives.

### Participants 

#### Nurses and nurse-midwives

Nurses and nurse-midwives who were working in the maternity ward of a single perinatal medical center and providing care for mothers of preterm infants were included regardless of their years of experience.

#### Sample size

The knowledge scores of healthcare workers on breastfeeding support were previously evaluated using the same study design [15]. The mean knowledge scores at pre-intervention and post-intervention were 20.2 points (Standard Deviation (SD): unknown, range 8–30) and 22.2 points (SD: unknown, range 7–34), respectively, with a significant difference (*p* < 0.05). Consequently, in the present study, the difference in the mean scores at pre-intervention and post-intervention was estimated to be 1.0 point. The SD of the knowledge scores was estimated to be 2.0. In the present case, the standardized effect size was calculated to be 0.5. The sample size was calculated to be 34 using G*Power version 3.1.9.2 software to determine statistical power. A power of 80%, a significance level of 0.05, and the paired t-test were the conditions applied when calculating the sample size. Considering a dropout rate of 20% during the survey, we planned to recruit 42 nurses and nurse-midwives to allow for attrition.

#### Maternal inclusion and exclusion criteria

The inclusion criteria were as follows: mothers (a) giving birth at a gestational age of less than 35 weeks in the participating hospital, (b) expressing breast milk to feed their own baby, (c) admitted to the maternity ward, (d) separated from their infant, and (e) with no history of breast surgery.

The maternal exclusion criteria included those who could not sufficiently recover after confinement and those whose infants were in critical condition with no recovery expected.

As for nutritional management of the preterm infant in the NICU, transition from tube feeding to oral feeding was considered at a corrected gestational age of 34 or more weeks [16]. On the assumption that mothers only needed to continue milk expression for 10 days after birth until they could breastfeed their infants directly, mothers who delivered an infant with a gestational age of less than 35 weeks were recruited.

### Data collection

#### Nurses and nurse-midwives

The lead researcher (RT) explained the purpose of this study (late June to July 2018). Three months before the education program (pre-1; late June 2018 to mid-August 2018), just before the education program (pre-2; mid-October 2018 to late November 2018), and 3 months after the education program (post-2; late January 2019 to mid-March, 2019), questionnaires regarding (a) *knowledge*, (b) *attitude*, and (c) *care implementation* were distributed and collected. Just after the education program (post-1; mid-October 2018 to late November 2018), the questionnaires regarding (a) *knowledge* and (b) *attitude* were collected. A *questionnaire to evaluate the education program* was also distributed to the participants who joined the education program with the above questionnaires at post-2, and then collected in a dedicated box. In this study, the period of education for nurses and nurse-midwives was from mid-October 2018 to late November 2018. The participants answered the questionnaires just before and after the education on the day they participated in the program. Therefore, pre-2 and post-1 were the same period.

#### Mothers

In the period before (June to mid-October 2018) and after (late November 2018 to May 2019) the education program for nurses and nurse-midwives, the lead researcher (RT) provided an explanation regarding this study to mothers who met the inclusion criteria in the maternity ward. The questionnaires were distributed to mothers who agreed to participate with the following contents: (a) *characteristics of mothers and infants*, (b) *mothers’ perception of nursing care for milk expression*, and (c) *a diary of milk expression for 10 days*. As for the diary, with respect to maternal milk expression after birth through the time when mothers agreed to participate in this study, the mothers recorded their milk expression retroactively based on the notes used for self-management during hospitalization. When the 10-day diary was complete, the mothers mailed it with the other filled-in questionnaires to the lead researcher (RT) in a reply envelope.

### Education program

#### Aims of the education program

A) To clarify the roles of nurses and nurse-midwives in early essential care for milk expression.
B) To understand the significance of early essential care for milk expression.
C) To know and implement appropriate early essential care for milk expression.

#### Contents of the education program

We developed and conducted a 60-min education program for nurses and nurse-midwives based on the Japanese guidelines [5] and a literature review [17]. The contents of care which should be enforced in an early postpartum period mainly in the maternity ward were extracted from the Japanese guidelines [5]. The education program was held face-to-face by the lead researcher (RT). The program was repeated 15 times to enable all the participants who had different work schedules to be able to attend (mid-October 2018 to late November 2018). The contents included the following topics (see Additional file 1):

A) Emotional support for mothers
B) Respect for mothers’ decision-making in relation to breastfeeding
C) Support for mothers, understanding the characteristics of breast milk, and significance of breastfeeding
D) Provision of information related to the necessity and methods of milk expression and assistance in the implementation
E) Mental support to mothers who cannot breastfeed and provision of information
F) Introduction of social resources related to breastfeeding

### Measurement tool

#### Nurses and nurse-midwives

##### Primary outcome

The *questionnaire regarding knowledge* was created based on the contents of the education program. It was composed of 20 items (see Additional file 1). When answering each item, the participants chose one from the following three options: “*Correct*”, “*Incorrect*”, and “*Unclear*”. The number of correct answers comprised the score.

##### Secondary outcome

The *questionnaire regarding attitude* was based on The Iowa Infant Feeding Attitude Scale [18]. It was composed of 10 items (see Additional file 1). Each item was scored on a five-point Likert scale (1 = *Strongly disagree*, 2 = *Disagree*, 3 = *Neither*, 4 = *Agree*, 5 = *Strongly agree*). The total scores of the 10 items were calculated.

The *questionnaire regarding care implementation* was based on the contents of the education program. It was composed of 10 items (see Additional file 1). Each item was scored on a five-point Likert scale (1 = *Never*, 2 = *Hardly ever*, 3 = *Some of the time*, 4 = *Most of the time*, 5 = *All of the time*).

The *questionnaire regarding evaluation of the education program* was based on a previous study [19] to evaluate its acceptability among the participants, demand, and practicality (see Additional file 1). Acceptability referred to the positive emotions of nurses and nurse-midwives to the education program. A questionnaire on acceptability was composed of the 4 items (Q1-Q4). Demand referred to necessity of the education program for nurses and nurse-midwives. A questionnaire on demand was composed of the 2 items (Q5, Q6). Practicality referred to utility value and usefulness of the education program for nurses and nurse-midwives. A questionnaire on practicality was composed of the 4 items (Q7-Q10). Each item was scored on a five-point Likert scale (1 = *Strongly disagree*, 2 = *Disagree*, 3 = *Neither*, 4 = *Agree*, 5 = *Strongly agree*). Furthermore, nurses and nurse-midwives were asked to describe the reason for their answer to each item in free text.

#### Mothers

A diary was created based on a previous report [17]. The diary included the following information: timing of initiation of milk expression, breast milk volume on the fourth and tenth days after birth, and frequency of milk expression for 10 days. As breast milk volumes on the fourth and tenth days after birth have been reported as being predictive of future milk production [5, 20, 21], we collected breast milk volumes at these time points. The lead researcher (RT) instructed the mothers to record the breast milk volume each time, regardless of the modality of expression. There was no method specified for measuring breast milk volume on the fourth day after birth because the timing of agreement to participate in this study was different for each mother. The lead researcher (RT) instructed the mothers to measure breast milk volume on the tenth day after birth using a calibrated syringe.

The *questionnaire regarding mothers’ perception of nursing care* was composed of two items: (a) care provided by nurses and nurse-midwives related to milk expression during hospitalization, and (b) impressions of care for milk expression by nurses and nurse-midwives. The mothers were asked to describe their perceptions using free text.

### Data analysis

Data from the questionnaire surveys were summarized using descriptive statistics [i.e., mean (standard deviation, SD) and median (interquartile range, IQR)] and used for statistical analysis. The Bonferroni method was applied to test for the correction of multiple hypotheses testing for time-series data. Therefore, the difference in the knowledge and attitude scores of nurses and nurse-midwives was considered to be significant if the *p*-value was less than 0.0125. Statistical analysis was performed using IBM SPSS Statistics version 24.0; Base and Advanced Statistics (IBM Japan, Tokyo, Japan). For descriptive data, illustrative quotes were extracted and reported.

## Results

### Nurses and nurse-midwives

#### Overview of participants

The lead researcher (RT) provided an oral and written explanation about this study to 44 nurses and nurse-midwives. Of the 41 nurses and nurse-midwives who agreed to participate, five did not take part in the education program. The reasons for not taking part were night shift only (2), maternity leave (1), sick leave (1), and poor physical condition (1) during the period of education. Therefore, the final participants were 36 nurse-midwives whose data were analyzed. The mean age of the participants was 31.8 years (range: 22–59, SD = 8.7). Their mean years of experience in the perinatal area was 6.5 years (range: 0–37, SD = 8.6). All of the 36 participants were nurse-midwives, three of whom were International Board Certified Lactation Consultants.

#### Knowledge

**Fig. 1:**
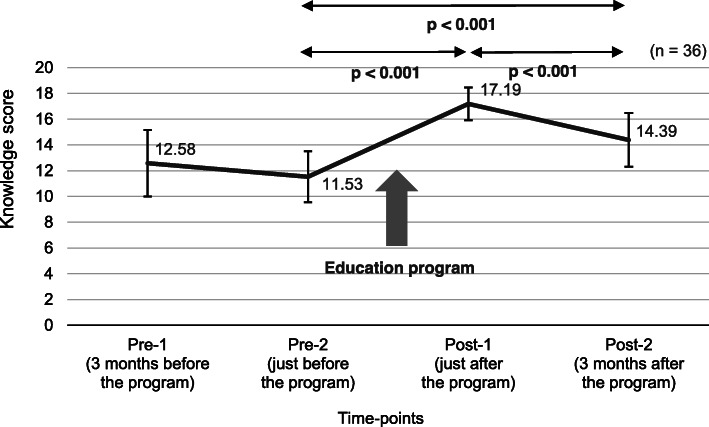
Knowledge scores (mean and SD) of nurse-midwives at four time-points

The mean knowledge scores were as follows: 12.58 points (range: 7–18, SD = 2.57, *n* = 33) at pre-1; 11.53 points (range: 5–15, SD = 1.98, *n* = 36) at pre-2; 17.19 points (range: 15–20, SD = 1.26, *n* = 36) at post-1; 14.39 points (range: 9–18, SD = 2.09, *n* = 36) at post-2 (Fig. 1).

The paired t-test showed no significant difference in the mean scores between pre-1 and pre-2. The mean score at post-1 was significantly higher than that at pre-2 (MD = − 5.67, *p* < 0.001, *n* = 36). The mean score at post-2 was also significantly higher than that at pre-2 (MD = − 2.86, *p* < 0.001, *n* = 36). The mean score at post-2 was significantly lower than that at post-1 (MD = 2.81, *p* < 0.001, *n* = 36).

#### Attitude

**Fig. 2:**
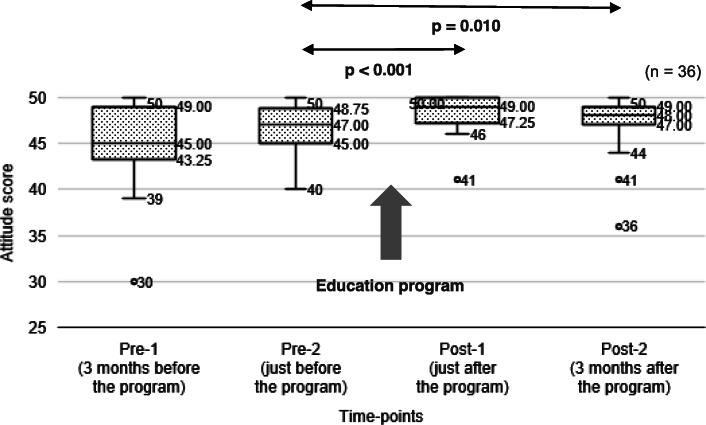
Attitude scores (median and IQR) of nurse-midwives at four time-points

The median scores and IQR of the nurse-midwives’ attitude on care at the four time-points are shown in Fig. 2.

The Wilcoxon signed-rank test showed no significant difference in the scores between pre-1 and pre-2. The scores at post-1 were significantly higher than those at pre-2 (z = − 4.05, *p* < 0.001, *n* = 36). The scores at post-2 were also significantly higher than those at pre-2 (z = − 2.57, *p* = 0.010, *n* = 36). There was no significant difference in the scores between post-1 and post-2.

#### Care implementation

The numbers of nurse-midwives who provided care related to milk expression for mothers of preterm infants over the last month were as follows: 29 nurse-midwives (90.6%, excluding four nurse-midwives whose questionnaires were unanswered) at pre-1, 27 nurse-midwives (75.0%) at pre-2, and 30 nurse-midwives (83.3%) at post-2.

The Wilcoxon signed-rank test for all items showed no significant difference in the scores of nurse-midwives’ care implementation between pre-1 and pre-2. However, for the following eight items, the scores of nurse-midwives’ care implementation at post-2 were significantly higher than those at pre-2: Q1, Q2, Q4, Q5, Q6, Q7, Q8, and Q10 (Table 1).

**Table 1:** Comparisons of care implementation between pre-2 (just before) and post-2 (after three months)

		(***n*** = 25)
Item	Z	***p -*** value
Q1 Provide care with a supportive attitude	− 2.24	**0.025**
Q2 Verify mothers’ motivation and intent	−2.65	**0.008**
Q3 Help mothers so that they will be motivated	−1.67	0.096
Q4 Explain about the benefits of breastfeeding	−2.80	**0.005**
Q5 Explain about the characteristics of the breast milk of mothers of preterm infants	−3.57	**< 0.001**
Q6 Explain about the necessity of milk expression	−2.50	**0.012**
Q7 Explain about the effect of initiating milk expression early and assist mothers in it	−3.09	**0.002**
Q8 Demonstrate the methods of milk expression	−2.45	**0.014**
Q9 Explain about the effect of frequent milk expression and encourage mothers to do so	−1.00	0.317
Q10 Explain about the effect of hand expression on lactogenesis 1	−2.88	**0.004**

#### Evaluation of education program

The response rate to the *questionnaire regarding evaluation of the education program* was 100%. The majority of the nurse-midwives “*Strongly agreed*” or “*Agreed*” with the all the questions related to the acceptability, demand, and practicality of the education program. The actual percentages of nurse-midwives who replied were as follows: “*Strongly agree*” or “*Agree*” 94.4% (34): Q1 (interest in care); 97.2% (35): Q2 (willingness to care); 97.2% (35): Q3 (understanding of care); 86.1% (31): Q4 (confidence in care); 100.0% (36): Q5 (necessity as skills); 97.2% (35): Q6 (necessity as practice in maternity wards); 97.2% (35): Q7 (valuable knowledge); 100.0% (36): Q8 (opportunity to look back on care); 97.2% (35): Q9 (adoption of care standards); 100.0% (36): Q10 (improvement of care).

One of the reasons for the willingness to care is expressed in this response by a nurse-midwife with less than 1 year of experience:

“*I understood that care for milk expression makes a difference in breast milk volume, and, my motivation has increased considering the benefits for mothers (e.g., increase in milk production).*”

The underlying description about the necessity of care learned in this education program as skills of midwives was expressed by a nurse-midwife with 10 years of experience as follows:

“*This is the care that we should learn because we have to provide this immediately after delivery.*”

One of the difficulties in adopting the standards of effective care was reflected in this response by a nurse-midwife with 4 years of experience:

“*The problem is that we sometimes cannot help mothers to express breast milk frequently (the delay of the first guidance related to the expression) because of the volume and order of priority of our work*.”

Additionally, there was a description that emphasized the balance between the mothers’ condition and evidence-based practice by another nurse-midwife with 4 years of experience as follows:

“*While considering the physical condition of mothers, I determine the frequency of milk expression*.”

### Mothers

#### Overview of mother participants

In the pre-intervention period, seven out of 10 mothers agreed to participate. In the post-intervention period, six out of six mothers agreed to participate. However, two did not return their questionnaires and diaries. Therefore, the remaining four mothers participated in this study (Table 2).

**Table 2:** Overview of participant mothers

		Mothers					Infants	
		Age range (years)	Parity	Delivery mode	Breast milk volume on 4th day (mL)	Breast milk volume on 10th day (mL)	Gestational age (w: weeks)	Approximate birth weight (g)
Before	A	≥ 35	Multipara	Vaginal	32.4	159	34w	2300
	B	≥ 35	Primipara	Cesarean	98	288	31w	1600
	C	≥ 35	Primipara	Cesarean	280	541	32w	1800
	D	25–34	Primipara	Cesarean	164	568	26w	900
	E	25–34	Primipara	Cesarean	516	887	34w	2000
	F	≥ 35	Primipara	Cesarean	100	350	33w	1700
	G	25–34	Primipara	Cesarean	344	508	34w	1400
After	H	≥ 35	Primipara	Cesarean	138	280	24w	500
	I	≥ 35	Primipara	Cesarean	54	185	28w	1000
	J	25–34	Primipara	Vaginal	190	280	34w	2100
	K	25–34	Multipara	Cesarean	170	675	31w	1100

#### Breast milk volume

The breast milk volumes of the individual mothers are shown in Table 2. The proportions of mothers whose milk volume reached 140 mL or more per day on the fourth day after birth were four out of seven (57.1%) in the pre-intervention period and two out of four (50.0%) in the post-intervention period. The proportions of mothers whose milk volume reached 500 mL or more per day on the tenth day after birth were four out of seven (57.1%) in the pre-intervention period and one out of four (25.0%) in the post-intervention period. The mean milk volume on the fourth day was 219.2 mL (range: 32.4–516, SD: 170.4) in the pre-intervention period and 138.0 mL (range: 54–190, SD: 60.0) in the post-intervention period. The mean milk volume on the 10th day was 471.6 mL (range: 159–887, SD: 236.0) in the pre-intervention period and 355.0 mL (range: 185–675, SD: 218.0) in the post-intervention period. The t-tests showed no significant difference in the mean breast milk volume between the groups in the pre-intervention and post-intervention periods on the fourth and tenth days after birth.

#### Timing of initiation of milk expression

**Fig. 3:**
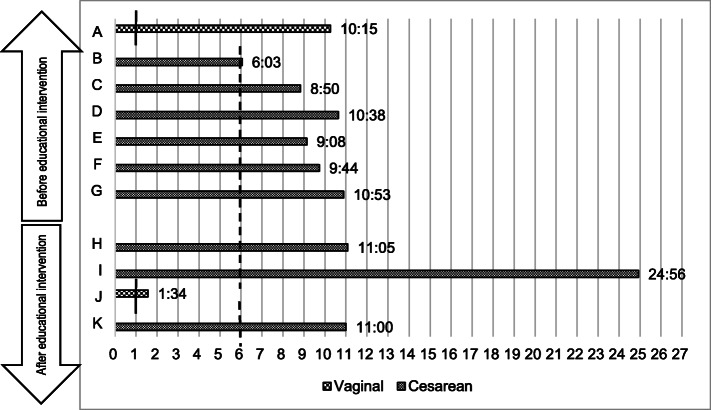
Number of hours before initiation of milk expression in mothers

The timing of initiation of milk expression after birth of each mother in the period before the education program for nurse-midwives is shown in Fig. 3. The solid vertical line indicates the appropriate criteria after vaginal delivery. The dotted vertical line indicates the appropriate criteria after cesarean section.

The mean number of hours before the initiation of milk expression in mothers was 9 h 21 min (range: 6 h 3 min to 10 h 53 min) in the pre-intervention period, and 12 h 8 min (range: 1 h 34 min to 24 h 56 min) in the post-intervention period. Even when the delivery mode is considered, there were no mothers who could initiate milk expression within the appropriate timing. However, mother B (before the education program for nurse-midwives) did initiate breast milk expression after about 6 h following her cesarean section, but not within 6 h. Mother J (after the education program for nurse-midwives) did initiate breast milk expression after about 1 h and a half following vaginal delivery, but this was not within 1 h.

#### Frequency of milk expression

**Fig. 4:**
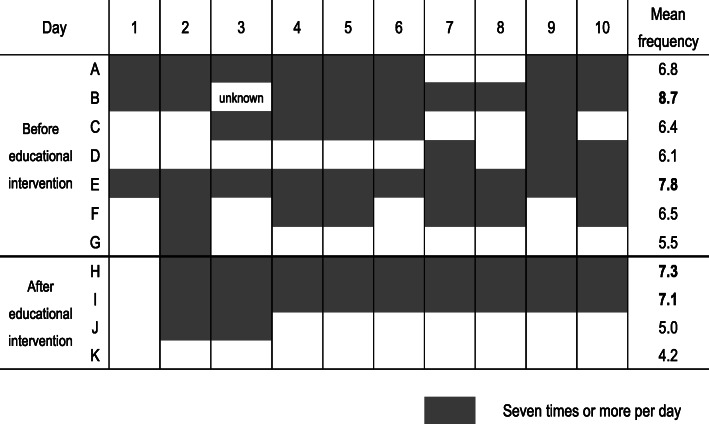
Frequency of milk expression per day for 10 days after birth by each mother

Except for the delivery day, the mean frequency of milk expression per day for 10 days after birth by each mother is shown in Fig. 4. The frequency of milk expression of Mother B was not available on day 3 (i.e., unknown) and was therefore not included in the average.

The mean frequency of milk expression per day was 6.8 times (range: 5.5–8.7, SD: 1.1) in the pre-intervention period and 5.9 times (range: 4.2–7.3, SD: 1.5) in the post-intervention period. The proportions of mothers whose mean frequency of milk expression per day was seven times or more were two out of seven (28.6%) in the pre-intervention period and two out of four (50.0%) in the post-intervention period.

#### Perception of nursing care for milk expression

Regarding the care for milk expression that mothers received during hospitalization, in the pre-intervention and post-intervention periods, the following forms of care were commonly provided: promoting blood circulation by warming and massaging the breasts, advising mothers to drink water, instructing mothers on methods of milk expression, assisting mothers in milk expression, and observing mothers’ breasts among others. The following forms of care were extracted only in the pre-intervention period: advising mothers to see their infant before milk expression, hearing about mothers’ previous breastfeeding experiences, and explaining to mothers about the symptoms of mastitis.

Regarding mothers’ perception of the care for milk expression by nurses and nurse-midwives, the following perceptions were commonly noted in the pre-intervention period:

happy with the empathic attitude of the nurses and nurse-midwives:

“*I was happy to be talked to [by nurses and nurse-midwives] that I was doing my best when I expressed just a little bit of breast milk for the first time*.”,

and increased motivation:

“*My motivation to express breast milk increased day by day because [the nurse] palpated [my breasts] every day and gave me appropriate advice*.”

One mother indicated a lack of nursing care only in the pre-intervention period:

“*I would like [the nurse] to have cared for me by massaging [the breasts], with a hot towel [hot compress] or by hand expression not only during the first three days but also the fourth or fifth days after delivery*.”

## Discussion

In this study, we examined the changes in the knowledge of appropriate care of nurse-midwives after implementing an education program focused on early essential care for breast milk expression among mothers of preterm infants.

### Changes in nurse-midwives by education

The education program improved the nurse-midwives’ knowledge and attitude on care related to breast milk expression among mothers of preterm infants until 3 months after the program. This improvement confirms a change in the primary outcome. It was previously reported that the knowledge and attitude of NICU nurses on providing breastfeeding support has been improved by educational intervention related to breastfeeding support [22]. However, in that previous study, improvement in nurses’ knowledge could not be sustained for 3 months after the education program. In the present study, implementation of the education program for only 60 min could improve and sustain the knowledge of nurse-midwives until 3 months after the program. Nevertheless, we needed to perform the education program 15 times to enable the 36 nurse-midwives who had different work schedules to be able to attend the program. Providing group guidance was found to be difficult because the participation of the nurse-midwives depended on their work schedule and ward workload of that day. With e-learning education programs, learners can learn anytime and anywhere [23] with reportedly no significant difference in knowledge acquisition from face-to-face or lecture-type learning [24]. Thus, it is necessary to consider and implement education programs using such learning methods.

The education program also significantly improved the nurse-midwives’ care implementation in most items until 3 months after the program. The knowledge of nurses on evidence-based practice was reportedly related to their attitude on evidence-based practice; thus, the knowledge and attitude of nurses affected the successful implementation of evidence-based practice [25]. Similarly in the present study, the increase in knowledge from the educational intervention is considered to improve the attitude and care implementation of the nurse-midwives.

However, the education program showed no improvement in the following item of care: Q9 Explain about the effect of frequent milk expression and encourage mothers to do so. One concern of nurse-midwives about adopting the standards of effective care was due to their work loads, indicating that the available manpower of busy nurse-midwives was a factor in how well care for milk expression could be implemented.

The lack of time and staff to support the milk expression of mothers has been reported to be a barrier against promoting milk expression in the early postpartum period [26]. Nevertheless, securing time and human resources necessary for implementing care related to milk expression was difficult to solve immediately with only the efforts of the individual nurses and nurse-midwives. Hence, it was difficult to promote continuous support of frequent milk expression for mothers only through educational intervention for nurse-midwives.

Another concern was that the perception of the mother’s condition was a factor in how well nurse-midwives could implement care for milk expression. It has previously been shown that obstetric nurses hesitated to provide care for milk expression to mothers with high blood pressure or mothers giving birth by cesarean section because of concerns about increased blood pressure and stress [26]. However, stimulation of the nipples induces the secretion of oxytocin which has positive effects including lowering of the blood pressure and levels of hormones related to stress, reducing pain associated with labor, and helping mothers acquire a maternal role by increasing social skills and reducing anxiety [27]. Therefore, stimulation of the nipples does not hinder the mental and physical recovery of mothers after birth. As stated in a previous study [26], it may have also been necessary to educate the nurse-midwives about the positive effects of oxytocin associated with milk expression to remove any hesitation in providing care.

### Limitations

The number of mothers was small, and this was the likely reason no differences in maternal milk expression were found. It was difficult to recruit many mother participants for the study. The sample size for mothers was not calculated beforehand because the primary outcome was *knowledge of appropriate care related to breast milk expression* of nurses and nurse-midwives to determine the benefit of the education program for nurses and nurse-midwives in this study. The researchers had assumed that seven to eight mothers per group could participate during the investigation period, considering the characteristic of the facility and the recruitment situation in the previous pilot study [10]. With reference to the data on milk volume of mothers obtained in this study, the ideal sample sizes required to show a significant difference between the groups in the pre- and post-intervention periods were 40 per group for breast milk volume on the fourth day after birth, and 61 per group for breast milk volume on the tenth day after birth. The change in the status of milk expression among mothers brought about by the education program for nurses and nurse-midwives is also very important. Therefore, if maternal milk volume is the primary outcome, it is necessary to conduct a survey at a facility with many cases of preterm births or at multiple facilities to meet this ideal sample size.

In this study, the method of data collection for maternal milk volume may not have been adequate for the assessment of the education program. If the mothers had the opportunity to breastfeed their infant directly in the NICU, we should have collected the data on the amount of breast milk the infants took. As the duration of hospitalization or lactation support of infants in the NICU varied between mothers, we needed to collect data related to factors affecting milk expression to interpret changes in maternal milk volume among the mothers following implementing education for nurse-midwives. Therefore, in future studies, it is necessary to develop a method for data collection that allows mothers to record and save their data related to milk expression simply and quickly, perhaps on a mobile phone application.

In this study, long-term assessments related to the status of milk expression of mothers were not conducted because factors other than the care provided in the maternity ward during the hospitalization of mothers might affect them, and these were not within the scope of this project. However, because exclusive breastfeeding for the first 6 months after birth was recommended [28], it is necessary to carry out long-term evaluation of this education program in the future considering factors other than the care provided in the maternity ward.

## Conclusions

We implemented an education program for nurse-midwives on early essential care for milk expression among mothers of preterm infants in the maternity ward. The education program was effective in helping nurse-midwives acquire *knowledge of appropriate care* and improve their *attitude on care* and *care implementation*. Moreover, most of the nurse-midwives favorably accepted the education program, felt it was necessary, and wanted to apply the standards of care learned from the program actively. These findings suggest that such an education program is required for nurse-midwife in-service education. The small number of mothers in the milk expression study limited the full determination of the benefits of the education program for nurse-midwives.

## Supplementary Information

**Additional file 1.** Education Program Package.

## Data Availability

The datasets and materials used during the current study are available from the corresponding author upon reasonable request.

## Abbreviations

IQR: Interquartile Range
MD: Mean Difference
mL: Millilitre
n: Number of cases
NICU: Neonatal Intensive Care Unit
Q: Question
SD: Standard Deviation
