# Supplementary material for: Implementing an education program for nurse-midwives focused on early essential care for breast milk expression among mothers of preterm infants
Source: Int Breastfeed J. 2021 Jun 26;16:47. doi: 10.1186/s13006-021-00395-z (PMC8235824; doi:10.1186/s13006-021-00395-z)
Supplement: Supplementary file 1 — Additional file 1. Education Program Package. [file 13006_2021_395_MOESM1_ESM.docx]

**Education Program**

| Pre-1 | *Questionnaire regarding knowledge*  *Questionnaire regarding attitude*  *Questionnaire regarding care implementation* |
| --- | --- |
| Pre-2 | *Questionnaire regarding knowledge*  *Questionnaire regarding attitude*  *Questionnaire regarding care implementation* |
| Education contents | 1. Emotional support for mothers 2. Understanding the mother’s mental situation 3. Supportive attitude such as receptive, empathetic, and considerate 4. Respect for mothers’ decision-making in relation to breastfeeding 5. Confirming the mother’s willingness to breastfeed 6. Increasing the mother’s motivation for breastfeeding 7. Support for mothers, understanding the characteristics of breast milk, and significance of breastfeeding 8. Characteristics of breast milk among mothers of preterm infants [1]   Encouraging the growth of the digestive tract among infants  Prevention of necrotizing enterocolitis and infection among infants  Enhancing digestion among infants  Prevention of chronic lung disease and retinopathy of prematurity among infants  Enhancing development of cognitive function among infants   1. Benefits of breastfeeding for mothers [2]   Promotion of uterine recovery after birth  Prevention of breast and ovarian cancer among mothers  Prevention of metabolic and cardiovascular diseases among mothers  Economic benefits   1. Provision of information related to the necessity and methods of milk expression and assistance in the implementation 2. Significance of milk expression for mothers   Feeding excellent nutrition to infants  Participation in medical care of infants  Maternal role   1. Importance of milk production in the early postpartum period   Milk output in the first week was significantly predictive of milk output in the sixth week [3].  Mothers with a low milk volume (< 140 ml/day) on the fourth day after birth were at risk of a lower milk production (< 500 ml/ day) on the sixth week after birth [4].   1. Mechanism of milk production and secretion   Lactogenesis stages  Endocrine control and autocrine control   1. Milk expression within one hour after birth [5, 6, 7]; milk expression within six hours for difficult cases such as cesarean section 2. Milk expression seven or more times per day [5, 8, 9] 3. Duration of pumping that exceeds 100 minutes per day [5] 4. Addition of hand expression to electric pumping more than five times a day until the onset of lactogenesis 2 if using an electric pump [9] 5. Methods of hand expression [10, 11] 6. Selection of a comfortable method for mothers 7. Mental support to mothers who cannot breastfeed and provision of information 8. Accepting the feelings of mothers about breastfeeding 9. Supporting the self-esteem and self-confidence of mothers 10. Introduction of social resources related to breastfeeding 11. Introduction of books on breastfeeding for mothers of preterm infants 12. Introduction of breast milk outpatients and midwifery homes |
| Post-1 | *Questionnaire regarding knowledge*  *Questionnaire regarding attitude* |
| Post-2 | *Questionnaire regarding knowledge*  *Questionnaire regarding attitude*  *Questionnaire regarding care implementation*  *Questionnaire regarding evaluation of the education program* |

**References**

1. Hirabayashi M. *Souzanji ni totteno bonyuu* [Breast milk for preterm infants]. In Mizuno K, editor. *Evidence ni motozuku souzanji bonyuuikuji manual* [Evidence-based breastfeeding manual for preterm infants]. Osaka: MEDICUS SHUPPAN, Publishers Co. Ltd.; 2015. 22-36.

2. Segawa M. *Bonyuuikuji no riten* [Benefits of breastfeeding]. In Japanese Association of Lactation Consultants, editors. *Bonyuuikujishien standard dainihan* [Standards for breastfeeding support]. 2nd ed. Tokyo: Igaku-Shoin Ltd.; 2015. 64-77.

3. Hill PD, Aldag JC, Chatterton RT, Zinaman M. Primary and secondary mediators’ influence on milk output in lactating mothers of preterm and term infants. Journal of Human Lactation. 2005;21(2):138-50.

4. Hill PD, Aldag JC. Milk volume on day 4 and income predictive of lactation adequacy at 6 weeks of mothers of nonnursing preterm infants. Journal of Perinatal & Neonatal Nursing. 2005;19(3):273-82.

5. Hopkinson JM, Schanler RJ, Garza C. Milk production by mothers of premature infants. Pediatrics. 1988;81(6):815-20.

6. Parker LA, Sullivan S, Krueger C, Kelechi T, Mueller M. Effect of early breast milk expression on milk volume and timing of lactogenesis stage II among mothers of very low birth weight infants: A pilot study. Journal of Perinatology. 2012;32(3):205-9.

7. Parker LA, Sullivan S, Krueger C, Mueller M. Association of timing of initiation of breastmilk expression on milk volume and timing of lactogenesis stage II among mothers of very low-birth-weight infants. Breastfeeding Medicine. 2015;10(2):84-91.

8. Hill PD, Aldag JC, Chatterton RT. Initiation and frequency of pumping and milk production in mothers of non-nursing preterm infants. Journal of Human Lactation. 2001;17(1):9-13.

9. Morton J, Hall JY, Wong RJ, Thairu L, Benitz WE, Rhine WD. Combining hand techniques with electric pumping increases milk production in mothers of preterm infants. Journal of Perinatology. 2009;29(11):757-64.

10. La Leche League International. The womanly art of breastfeeding. 6th revised ed. Illinois: La Leche League International; 1997. 113-49.

11. UNICEF/WHO. Breastfeeding management and promotion in a baby-friendly hospital: an 18-hour course for maternity staff. New York: UNICEF; 1993. 91-100.

***Questionnaire regarding knowledge***

| Item (answer) |
| --- |
| **Q1** Nurses must not mention about breast milk expression because of the complex psychological state of mothers after birth. (Incorrect) |
| **Q2** Emotional support for mothers is important in the care related to milk expression. (Correct) |
| **Q3** Nurses should verify the motivation and intent of mothers after providing information about breastfeeding. (Incorrect) |
| **Q4** Breastfeeding is effective in preventing metabolic and cardiovascular diseases in mothers. (Correct) |
| **Q5** Breast milk is effective in preventing chronic lung disease and retinopathy of prematurity in infants. (Correct) |
| **Q6** Breast milk expression is essential for mothers of preterm infants to participate in the medical care of  infants. (Correct) |
| **Q7** Mothers with a milk volume of less than 140 ml/day on the fourth day after birth are at risk of lower milk production. (Correct) |
| **Q8** Mothers with a milk volume of 350 ml or more per day at around the second week after birth tend to maintain breast milk volume easily. (Incorrect) |
| **Q9** The period from the 16th week of pregnancy through the second day after birth is called lactogenesis 1. (Correct) |
| **Q10** Milk production increases and is established on and after the ninth day after birth. (Incorrect) |
| **Q11** Breast milk which is produced in lactogenesis 2 is called mature milk (*seinyu*). (Incorrect) |
| **Q12** Autocrine control means that breast milk is produced by the action of hormones such as prolactin. (Incorrect) |
| **Q13** Frequent breast milk expression is essential so as not to lower the blood levels of prolactin after birth. (Correct) |
| **Q14** Breast milk volume is directly proportional to a longer time between birth and the initiation of breast milk expression. (Incorrect) |
| **Q15** Mothers who initiate breast milk expression less than one hour after birth produce significantly more milk in the first week after birth. (Correct) |
| **Q16** Mothers expressing milk six or more times a day can obtain a high milk volume. (Incorrect) |
| **Q17** Optimal milk production is associated with the duration of pumping that exceeds 100 minutes per day. (Correct) |
| **Q18** It is better to use hands instead of a breast pump until the beginning of lactogenesis 2. (Incorrect) |
| **Q19** Nurses should recommend a breast pump after discharge so that mothers can obtain significantly more milk. (Incorrect) |
| **Q20** Nurses must not mention breastfeeding to mothers who cannot breastfeed for medical reasons. (Incorrect) |

***Questionnaire regarding attitude***

| Item |
| --- |
| **Q1** Breast milk is the most ideal nutrition for preterm infants. |
| **Q2** Breastfeeding deepens the relationship between preterm infants and mothers. |
| **Q3** Breast milk expression is one of the most important maternal roles. |
| **Q4** I am interested in care related to milk expression for mothers of premature infants. |
| **Q5** I want to provide care related to milk expression for mothers of preterm infants positively. |
| **Q6** It is better to initiate breast milk expression as soon as possible after birth. |
| **Q7** It is better to maintain breast milk expression as often as possible after birth. |
| **Q8** It is better for mothers to obtain breast milk volume as much as possible before discharge from hospital. |
| **Q9** Care related to milk expression puts pressure on mothers. |
| **Q10** Care related to milk expression is not so essential for mothers of preterm infants. |

Each item was scored on a five-point Likert scale (1 = *Strongly disagree*, 2 = *Disagree*, 3 = *Neither*, 4 = *Agree*, 5 = *Strongly agree*).

**Note:** The scores of Q9 and Q10 were inverted as follows: 5 = *Strongly disagree*, 4 = *Disagree*, 3 = *Neither*, 2 = *Agree*, 1 = *Strongly agree*.

***Questionnaire regarding care implementation***

| Item |
| --- |
| **Q1** I provided care to mothers with a supportive attitude such as receptive, empathetic and considerate attitude. |
| **Q2** I verified mothers’ motivation and intent to breastfeed. |
| **Q3** I helped mothers so that they would be motivated to breastfeed. |
| **Q4** I clearly explained to mothers about the benefits of breastfeeding. |
| **Q5** I clearly explained to mothers about the characteristic of breast milk of mothers of preterm infants. |
| **Q6** I explained to mothers about necessity of breast milk expression for mothers and infants. |
| **Q7** I explained to mothers about the effect of initiating breast milk expression early after birth and assisted them in it. |
| **Q8** I clearly demonstrated the methods of breast milk expression to mothers. |
| **Q9** I explained to mothers about the effect of frequent breast milk expression and encouraged them to do so. |
| **Q10** I explained to mothers about the effect of hand expression in lactogenesis 1. |

Each item was scored on a five-point Likert scale (1 = *Never*, 2 = *Hardly ever*, 3 = *Some of the time*, 4 = *Most of the time*, 5 = *All of the time*).

**Note**: Care implementation for the past month was answered for these items.

***Questionnaire regarding evaluation of the education program***

| Item |
| --- |
| **Q1** I became (more) interested in care related to breast milk expression for mothers of preterm infants. |
| **Q2** I was (more) willing to provide care related to breast milk expression for mothers of preterm infants. |
| **Q3** I obtained (more) understanding of care related to breast milk expression for mothers of preterm infants. |
| **Q4** I acquired (more) confidence in providing care related to breast milk expression for mothers of preterm infants. |
| **Q5** I think that we should learn these as the skills of nurses and nurse-midwives. |
| **Q6** I think that these are essential for nursing practice in the maternity wards to which a NICU is attached. |
| **Q7** I acquired valuable knowledge of providing care related to breast milk expression for mothers of preterm infants through this program. |
| **Q8** I had an opportunity to look back on the care related to breast milk expression for mothers of preterm infants through this program. |
| **Q9** I want to adopt the standards of effective care related to breast milk expression (timing of initiating breast milk expression, frequency, breast milk volume etc.) by positively applying knowledge acquired through the education program. |
| **Q10** I think that this program is valid to improve the quality of care related to breast milk expression for mothers of preterm infants. |

Each item was scored on a five-point Likert scale (1 = *Strongly disagree*, 2 = *Disagree*, 3 = *Neither*, 4 = *Agree*, 5 = *Strongly agree*).

**Note**: Q1-Q4: acceptability; Q5, Q6: demand; Q7-Q10: practicality
